# Silicon Self-Switching Diode (SSD) as a Full-Wave Bridge Rectifier in 5G Networks Frequencies

**DOI:** 10.3390/s22249712

**Published:** 2022-12-11

**Authors:** Tan Yi Liang, Nor Farhani Zakaria, Shahrir Rizal Kasjoo, Safizan Shaari, Muammar Mohamad Isa, Mohd Khairuddin Md Arshad, Arun Kumar Singh

**Affiliations:** 1 Faculty of Electronic Engineering Technology, Universiti Malaysia Perlis (UniMAP), Arau 02600, Perlis, Malaysia; 2 Advanced Communication Engineering, Centre of Excellence (ACE), Universiti Malaysia Perlis (UniMAP), Kangar 01000, Perlis, Malaysia; 3 Department of Electronics and Communication Engineering, Punjab Engineering College (Deemed to Be University), Sector-12, Chandigarh 160012, India

**Keywords:** microwave rectifier, sustainable energy, microwave detector, silicon on insulator

## Abstract

The rapid growth of wireless technology has improved the network’s technology from 4G to 5G, with sub-6 GHz being the centre of attention as the primary communication spectrum band. To effectively benefit this exclusive network, the improvement in the mm-wave detection of this range is crucial. In this work, a silicon self-switching device (SSD) based full-wave bridge rectifier was proposed as a candidate for a usable RF-DC converter in this frequency range. SSD has a similar operation to a conventional pn junction diode, but with advantages in fabrication simplicity where it does not require doping and junctions. The optimized structure of the SSD was cascaded and arranged to create a functional full-wave bridge rectifier with a quadratic relationship between the input voltage and outputs current. AC transient analysis and theoretical calculation performed on the full-wave rectifier shows an estimated cut-off frequency at ~12 GHz, with calculated responsivity and noise equivalent power of 1956.72 V/W and 2.3753 pW/Hz^1/2^, respectively. These results show the capability of silicon SSD to function as a full-wave bridge rectifier and is a potential candidate for RF-DC conversion in the targeted 5G frequency band and can be exploited for future energy harvesting application.

## 1. Introduction

In recent years, the 5th generation (5G) technology has attracted the interest of researchers as 4G has matured, therefore only little improvement and several fresh carrier proficiencies can be expected [1,2]. This 5G technology has contributed to the rapid spread of the Internet of Things (IoT), where it wirelessly connects a huge number of physical items such as wearable devices [3], smart home sensors [4], industrial sensors, and agricultural sensors to deliver services such as healthcare, smart industry, and smart agriculture [5]. In the construction of the IoT environment, energy limitation was revealed as one of the huge challenges faced by the IoT application, where most of the IoT application, especially in portable and wearable devices, require long last battery and energy [6].

The application of green energy technologies for energy harvesting (EH) are important tools in prolonging the lifetime of the IoT, extending the lifetime of wireless energy-constrained networks by avoiding the need for hard-wiring or replacing mobile batteries [7,8]. Conventional energy harvesting systems extract energy from the environment via the sun [9], wind, vibration, thermoelectric effects [10], or other physical phenomena, which are less applicable in the scenario where access to external energy sources are limited [11,12]. With the vast availability of radio frequency (RF) transmitters (mobile base stations, TV and radio stations, Wi-Fi routers, etc.), ambient RF transmissions were a promising approach with the advantage of transporting energy and information [13]. 

**Figure 1:**
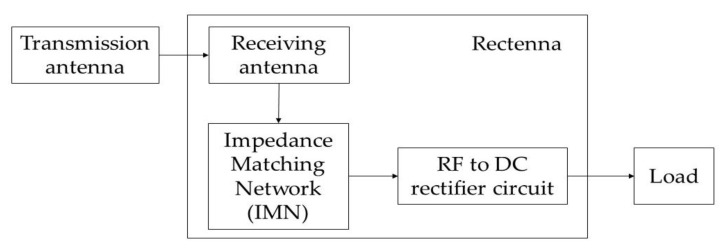
RF energy harvesting system [14].

Figure 1 shows the illustration of an RF energy harvesting system, which consists of a transmission antenna, receiving antenna, impedance matching network (IMN), rectifier circuit, and load [14]. The RF signals are first transmitted by the transmission antenna and then received by the receiving antenna. The IMN adjusts the impedance between the receiving antenna and the rectifier circuit. In the absence of IMN, all the energy received by the system will be reflected, causing a decreased amount of rectified output current obtained by the load [15]. The RF signals are then transferred from the IMN to the rectifier circuit for radio frequency waves to direct current (RF-DC) conversion, before being supplied to the load. In 5G networks, the RF range is in the millimeter wave (mm-wave) band, which includes frequencies ranging from 3 GHz to 300 GHz [16]. These mm-wave band operation frequencies as presented by the 3rd Generation Partnership Project (3GPP) consists of frequency range 1 (FR1) and frequency range 2 (FR2), operating at a frequency range of 450 MHz to 6 GHz and 24.25 GHz to 52.6 GHz, respectively [17]. 

The motivation of this research is to accommodate the RF-DC energy harvesting system in the 5G networks frequencies by providing an alternative device for an efficient RF-DC conversion. Most widely used and marketed rectifiers in the microwave region is the Schottky diode, where it brings an advantage of a higher turn-on voltage compared to a conventional pn junction diode. Other silicon-based bridge rectifiers focusing on 5G networks frequencies are also shown in Table 1, and most are seen focusing on the lower FR1 of the 5G spectrums. The fabrication process steps in all existing silicon-based rectifiers consists of complex fabrication steps, with the involvement of multiple doping layers, and multiple steps of lithography process for device patterning. 

In this work, a full-wave bridge rectifier for sustainable 5G network application is proposed by using a silicon-based self-switching device (SSD) [15]. SSDs have received attention from researchers worldwide as they have been reported to effectively function as zero-bias RF detectors [8,18]. The rectification property of the SSD is similar to a pn junction diode, with simplicity in fabrication process where it can be simply realized by one-step lithography process and chemical etching, and does not involve junctions, doping, or third gate terminal [19]. In SSD, a pair of L-shaped trench are etched between two electrodes, resulting in depletion region in between the etched region (air) and silicon [20]. Depending on the bias given, the thickness of this depletion region can be controlled to open and close the channel, which created a non-linear response of the IV characteristic, similar to a conventional diode. In addition, the planar configuration of the SSD where all the electrical contacts are in the same plane will result in a smaller parasitic capacitance compared to other diodes [21,22].

**Table 1:** The results reported from previous works on bridge rectifier.

Rectifier	Frequency	Fabrication Process Steps	Simulation/ Fabrication	References
Schottky barrier diode Bridge rectifier	2.45 GHz	-	Simulation	[23]
Schottky barrier diode bridge rectifier	100 MHz 2.45 GHz	-	Simulation	[24]
NMOS bridge rectifier	13.56 MHz	-	Simulation	[25]
SOI CMOS bridge rectifier	2.45 GHz	-	Simulation	[26]
Integrated 4H-Silicon Carbide diode bridge rectifier	-	etching, annealing, patterning and metal deposition	Fabrication	[27]
GaAs Schottky diodes bridge rectifier	-	heavily doping, lightly doping, etching and metal deposition	Fabrication	[28]
Oxide TFT Rectifiers	-	multi dry etching, multi deposition	Fabrication	[29]

**Figure 2:**
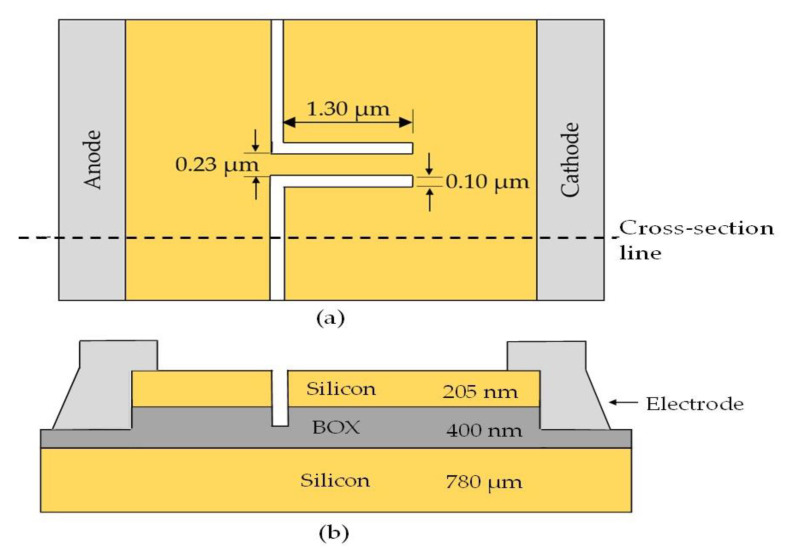
(**a**) Structural parameters of an SOI-based SSD, showing three main control factors: L, W, and Wt, and (**b**) the cross-section of the device [32].

SSD as a full wave bridge rectifier has only been evaluated on graphene-based SSD targeting a higher Terahertz frequency region. However, the feasibility issues and usefulness of a graphene based rectenna in ambient RF harvesting is minimal [30] and patterning issues of graphene for integration with electronic devices in the realization of monolithic-integration microwave integrated circuits (MMIC) is a big hurdle to overcome [31]. Therefore, in this paper, the capability of a silicon-based SSD to function as a full-wave bridge rectifier to convert RF to DC is examined and concluded for better integration with current CMOS technology, and for future implementation in a RF-DC energy harvesting system. Furthermore, an optimized structure of a silicon based SSD is shown in Figure 2, which has been reported in [32] and shows a cut-off frequency at around 6.50 GHz, where it shows a promising capability to operate in the FR1 of the 5G spectrums [32], which carried most of the traditional cellular mobile communications traffic [33,34]. In addition, experimental research on silicon-based SSD shows no leakage and breakdown voltage from 0 to −5 V bias which is an advantage to a rectifier device [35]. 

## 2. Materials and Methods

**Figure 3:**
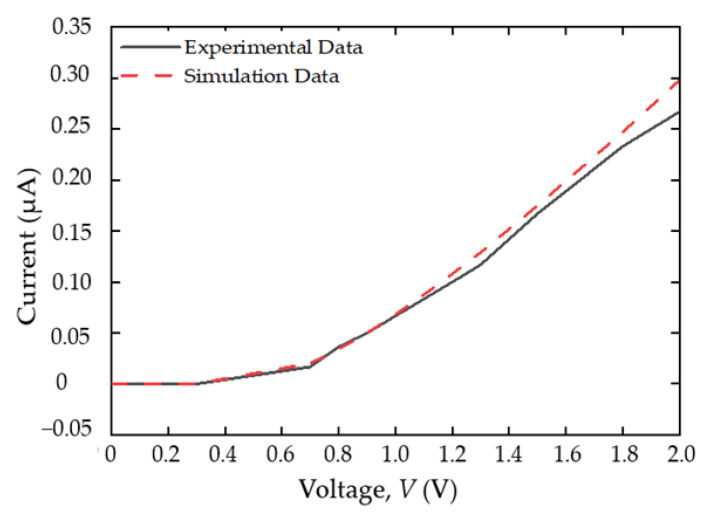
Comparison of IV characteristics between simulation and experimental data of Farhi et al. [32,35].

Prior to the design of the full-wave bridge rectifier, a validation process on the physical models and material parameters of the SSD defined in the device simulator (ATLAS Silvaco) were performed by comparing the electrical characteristics to the experimental results of a silicon SSD structure from [35]. A p-type silicon substrate SSD with doping concentration of 2.45 × 10^16^ cm^−3^ were defined with interface charge density of 3.16 × 10^11^ cm^−2^ along the channel [35,36]. In addition, physical models such as Klaassen’s unified low-field mobility model, the Watt model, Auger recombination, and the energy balance transport were implemented in the simulation to imitate the electron transport mechanism of the real device [32]. As shown in Figure 3, the I-V characteristics of the simulated and experimental data [35] were in good agreement, which validated the models and parameters used in the simulation. 

**Figure 4:**
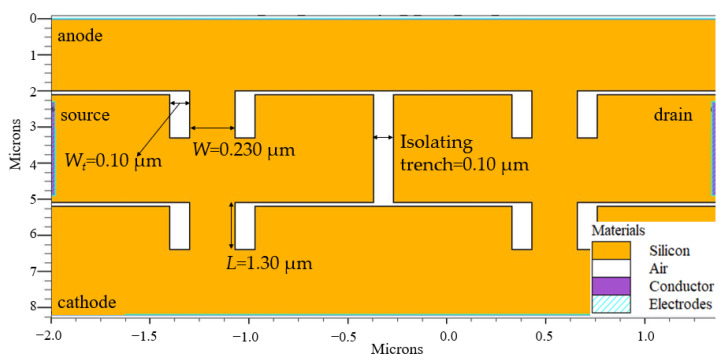
The structural parameters of the SSD full wave bridge rectifier.

**Figure 5:**
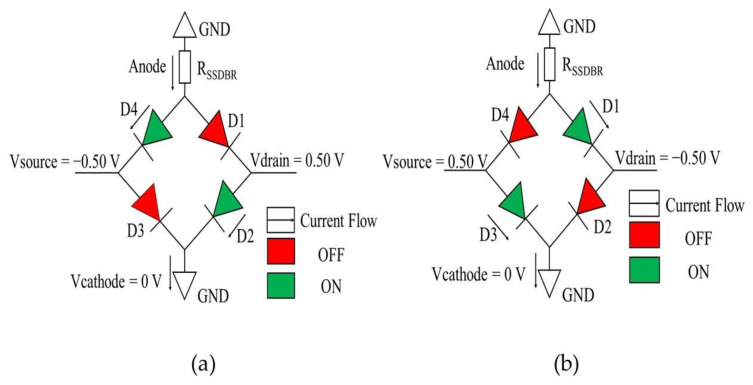
The equivalent circuit for different biasing applied to the bridge rectifier in (**a**) positive cycle and (**b**) negative cycle.

A full-wave bridge rectifier was then designed using the optimized structure of silicon-based SSD as shown in Figure 2. The full-wave bridge rectifier, as shown in Figure 4, consists of four electrodes; drain, source, anode, and cathode at the right, left, top, and bottom of the device, respectively. An isolating trench with a width of 0.10 µm was also etched between the SSD’s two series connection. This trench separates the SSDs in the series network. For ease of understanding, the mechanism of this full-wave bridge rectifier is explained as the equivalent diode-based circuit diagram (representing the SSD) in Figure 5. 

Two different current flows occurred when different polarities of the AC input signals are applied to the source and drain. When the source and drain are applied −0.50 V and 0.50 V, respectively, the D2 and D4 are in forward bias and D1 and D3 are in reverse bias, as shown in the circuit diagram of Figure 5a. The cathode is applied 0 V bias to act as a ground. Figure 5b is the diagonal opposite operation of Figure 5a when the polarities of the drain and source are changed to −0.50 V and 0.50 V, respectively, while keeping the cathode at 0 V. A series resistance, *R*_SSDBR_ of 615 kΩ were also defined in the simulation to imitate real SSD resistance [35]. 

To examine the current output response of the device, DC and AC transient analyses were conducted on the device using ATLAS Silvaco device simulator. Based on the I-V characteristic obtained from the DC analysis, the noise equivalent power (NEP) and responsivity were calculated, which indicates the minimal detectable power per square root bandwidth and the rectification performance of the full-wave bridge rectifier, respectively. Furthermore, to imitate the RF sinusoidal waves input in different frequencies, an AC input signal were given at the source and drain from 1 to 12 GHz frequency and the DC output current responses were recorded. The cut-off frequency was then evaluated by analyzing the DC output current response to observe the maximum frequency that the full-wave bridge rectifier can rectify. 

## 3. Results and Discussion

### 3.1. The Mechanism of the SSD Full-Wave Bridge Rectifier

**Figure 6:**
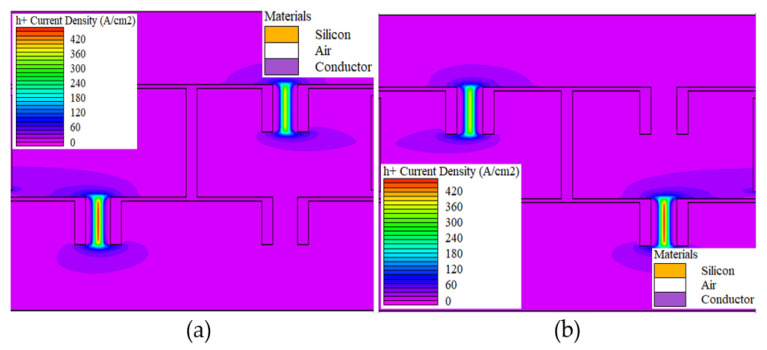
(**a**,**b**) The hole current density of the SOI SSD full wave bridge rectifier.

Figure 6 shows the hole current density that flowed across the SSD, representing the biasing condition of the conventional full-wave bridge rectifier obtained from the Silvaco TonyPlot 2D software version 5.0.22.R by Silvaco company from Santa Clara, California. The biasing conditions and current flows were similar to the mechanism explained in diode-based circuit diagram of Figure 5a,b, where in the positive cycle of Figure 6a, the SSDs representing D1 and D2 are in forward bias, while no current flow in D3 and D4, which indicates reverse bias. In negative cycle of Figure 6b, the SSDs representing D3 and D4 are in forward bias, while D1 and D2 are in reverse bias.

### 3.2. I-V Characteristics of the SSD Full-Wave Bridge Rectifier

**Figure 7:**
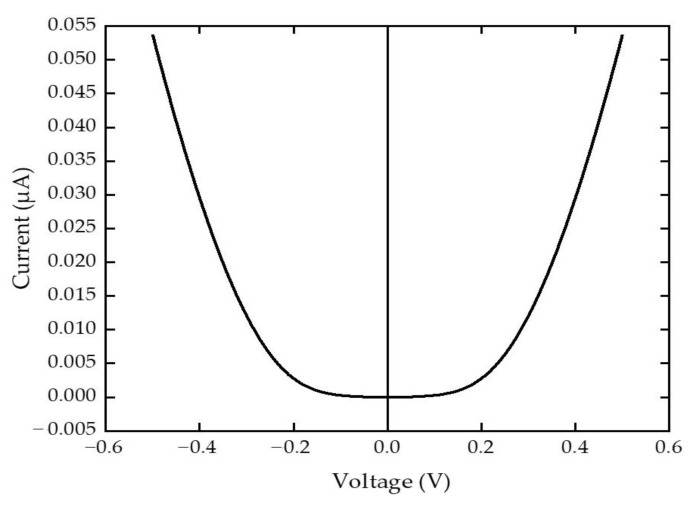
I-V characteristics of the SSD full wave bridge rectifier.

Figure 7 shows the I-V characteristic of the SSD with varied DC bias voltages from −0.5 V to 0.5 V. From the graph, it can be observed that the output currents of the full-wave bridge rectifier are always positive regardless of the polarity of the voltage applied in the source and drain. The highest output current achieved from both positive and negative cycle was 0.05361 µA at V = 0.5 V and V = −0.5 V, respectively, which is higher than the reported output current value of 0.04274 µA at V = 0.5 V in single optimized SSD structure [32]. When source and drain were applied with voltage, some of the holes and electrons diffused through the isolating trench causing the concentration of holes and electrons to increase. This could cause threshold voltage drop in the device with increased forward current behavior. This brings an advantage of a faster turn-on transition in between both negative and positive polarities of the sinusoidal RF waveform in full wave rectifier configuration. 

Moreover, the noise equivalent power (NEP) and responsivity was calculated from the I-V characteristics to determine the rectification performance of the full-wave bridge rectifier using the equation of:

(1) $$\beta =2R_{s}\gamma$$

where *β* is the responsivity, *R_s_* is the source impedance and *γ* is the curvature coefficient [28]. The *β* value obtained was 1956.72 V/W with *R_s_* = 50 Ω and *γ* = 19.5672 V^−1^. This responsivity value is better than the holes of SSD full-wave bridge rectifier reported in graphene which was reported at 1571 V/W [37]. 

The NEP of the device was also calculated to determine the minimal detectable power per square root bandwidth by using the equation of:

(2) $$NEP=\frac{\sqrt{4kTR}}{\beta }$$

where *k* is the Boltzmann constant, *R* is the zero-bias resistance and *T* is the temperature [38,39]. This parameter may show the measure of the sensitivity in a detector system in a device level. The NEP obtained was 2.3753 pW/Hz^1/2^ with *R* = 1.3036 kΩ and *T* = 300 K, which also shows better performance compared to SSD full-wave bridge rectifier’s NEP in graphene, which is 18.2 pW/Hz^1/2^ [37]. 

### 3.3. Rectification of the SSD Full-Wave Bridge Rectifier

**Figure 8:**
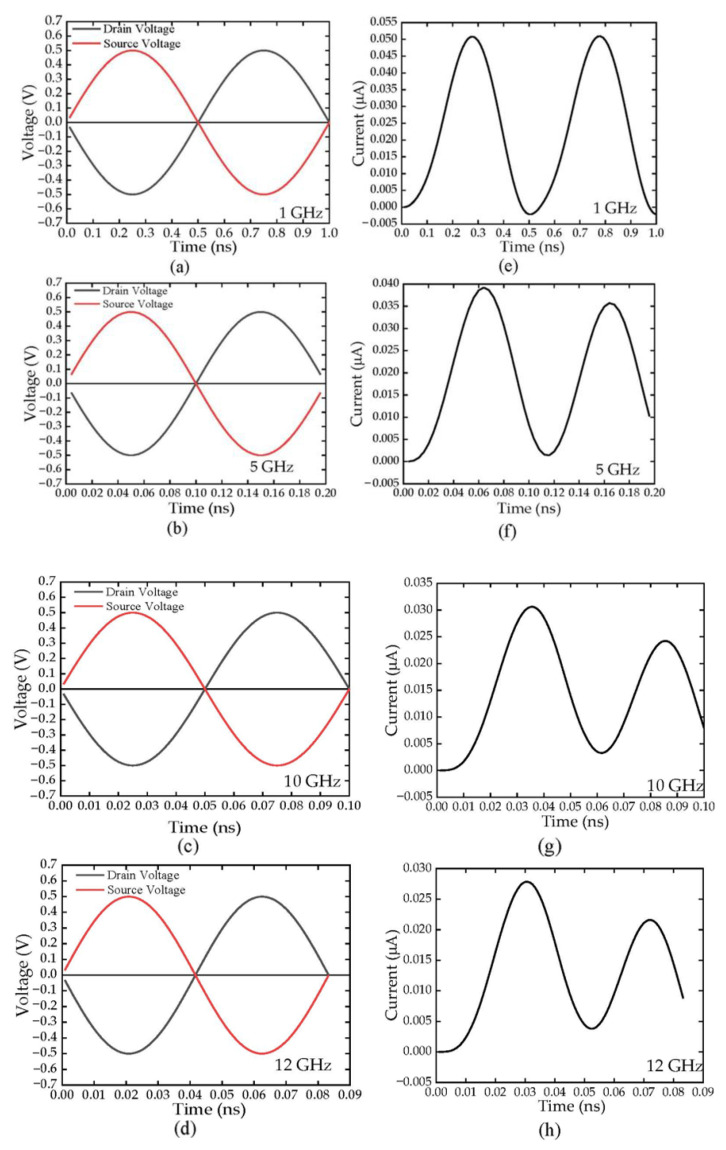
The AC signal inputs at (**a**) 1 GHz, (**b**) 5 GHz, (**c**) 10 GHz, and (**d**) 12 GHz with the rectified output DC current in (**e**–**h**), respectively.

Figure 8a–d shows simulated AC input signals with an amplitude of 0.5 V were inserted between drain and source to imitate the RF wave into the devices, at frequency of 1, 5, 10, and 12 GHz, respectively. The respective current output response of the rectifier were observed at the anode, and are shown in Figure 8e,h. As can be observed, at 1 GHz, the output responded well to the input signal with high current output values both in positive and negative polarity of the drain voltage. Additionally, in higher frequency, the amplitude of the DC outputs was observed lower with reduced peak in opposite polarity at second peak. Since energy of radiation is inversely proportional to its wavelength, it can be observed that the amplitude of the output current decreased in lower wavelength (higher frequencies). The ripple is however reduced at higher input switching frequency because of the faster transition between the positive and negative input cycles. 

### 3.4. Cut-Off Frequency of the SSD Full-Wave Bridge Rectifier

**Figure 9:**
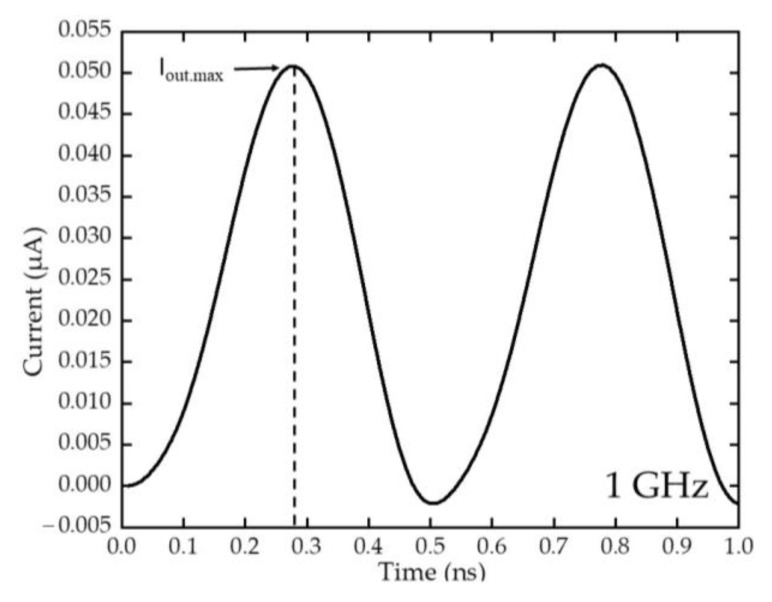
Maximum output current, I_out.max_ from the rectified output DC current.

**Figure 10:**
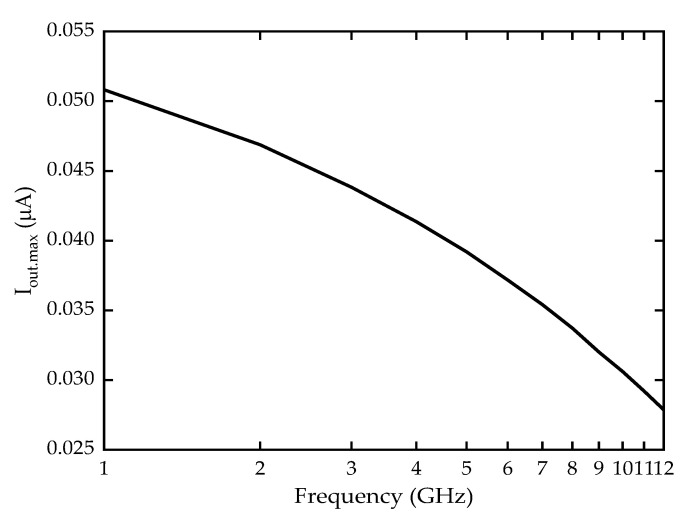
Maximum output current I_out.max_ in various frequencies.

The maximum rectified output current, I_out.max_ from the AC signal analysis were obtained, as shown in Figure 9, and the I_out.max_ from 1GHz to 12 GHz were plotted in Figure 10. As can be observed, the I_out.max_ can be seen decreased when the frequency increased, with the highest I_out.max_ value observed at 50.82 nA with 1 GHz frequency input. The value continues to decrease to around 30.60 nA at 10 GHz, because of the reduced energy in higher frequency as explained in Section 3.3 [40]. This current output value at 10 GHz is comparable to previous energy harvesting research by using piezo-triboelectric hybrid nanogenerator which has obtained a 30 nA output as an operable current output [41]. Additionally, since the device is targeting the FR1 frequencies at sub-6 GHz frequency, the current output at 6 GHz is being focused which shows 37 nA of usable current output.

**Figure 11:**
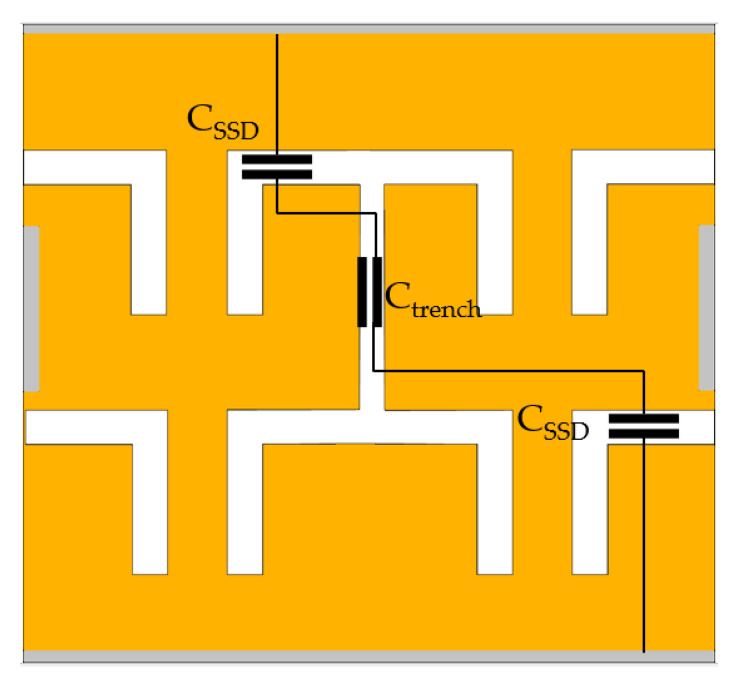
Capacitance estimation of SSDBR.

To verify the cut-off frequency of the bridge rectifier obtained from the device simulator, a theoretical calculation of the cut-off frequency was conducted using Equation (3) [42,43,44]:

(3) $$f_{c}=\frac{1}{2\pi R_{SSDBR}C_{SSDBR}}$$

where *R_SSDBR_* is the resistance of the SSD full-wave bridge rectifier and *C_SSDBR_* is the calculated capacitance of the full-wave bridge which is the combination of two SSDs channel capacitances, *C_SSD_* and trench capacitance, *C_trench_*, as illustrated in Figure 11. In one complete cycle, the currents will be passing through two diagonally connected SSDs. Additionally, at high frequencies, holes are diffused through the trench causes induced extrinsic capacitance, which also decreases the overall output current as observed in Figure 8a–g. 

**Figure 12:**
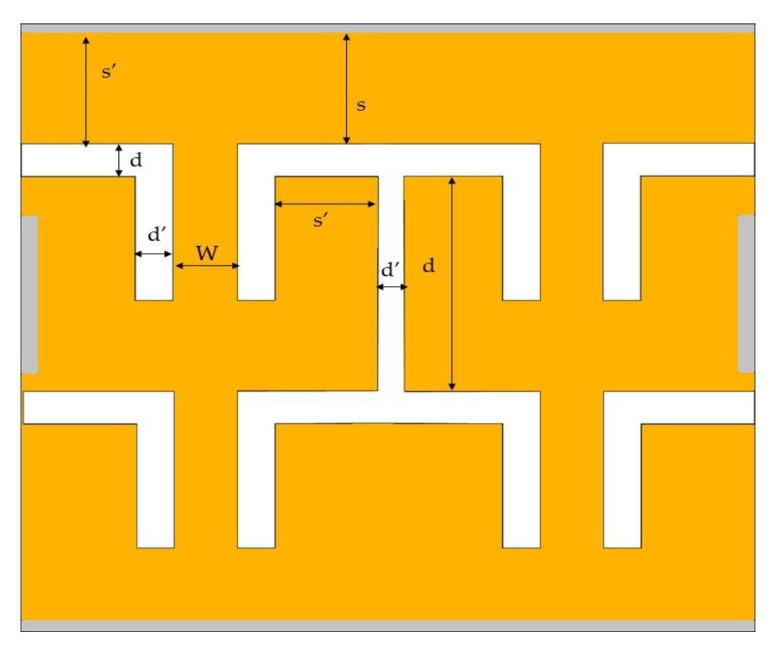
Structural parameters for estimation of channel capacitances, *C_SSD_* and trench capacitance, *C_trench_*.

The capacitances were calculated by using conformal mapping techniques [45,46] which involves the equations shown in Equations (4)–(6) below:

(4) $$\frac{C_{v}}{\varepsilon_{r}\varepsilon_{o}}=\frac{1}{\pi }\left(8\frac{s}{d}\right),s\geq d$$

(5) $$\frac{C_{v}}{\varepsilon_{r}\varepsilon_{o}}=\frac{\pi }{ln\left[4\left(d/s\right)\right]}$$

(6) $$C_{H}=4\pi \varepsilon_{r}\varepsilon_{o}\left(\frac{\pi }{\ln\left(\frac{4}{k_{1}}\right)}\right),k_{1}=\frac{a}{b}\sqrt{\frac{c^{2}-b^{2}}{c^{2}-a^{2}}},a=\frac{W}{2},b=\left(\frac{W}{2}+d^{\prime }\right),c=\left(\frac{W}{2}+d^{\prime }+s^{\prime }\right)$$

where the relative permittivity, *ε_r_* = 6.34 with conformal mapping parameters as shown in Figure 12 and Table 2. This *ε_r_* value is the average value of dielectric constant of silicon (*ε_r_* = 11.68) and air (*ε_r_* = 1).

The calculated *C_SSD_* was 1.785 × 10^−16^ *F* using Equations (4) and (6), while *C_trench_* = 3.6701 × 10^−17^ *F* using Equations (4) and (5). The total *C_SSDBR_* obtained from the addition of *C_SSD_* and *C_trench_* is *C_SSDBR_* = 2.1838 × 10^−17^ *F* with 0.205 µm thickness active device layer. The cut-off frequency calculated using Equation (3) shows cut-off value at 12 GHz which is well in accordance with the simulated results showed in Figure 10. 

## 4. Conclusions

A full-wave bridge rectifier based on silicon self-switching device (SSD) were proposed in this work. The simulation result and analyses show a usable output current value around 30.60 nA at 10 GHz frequency, with cut-off at around 12 GHz. The results obtained in this research proves the capability of the silicon-based SSD full-wave bridge rectifier to function as the RF-DC converter in the FR1 spectrum of the 5G networks. Furthermore, the calculated responsivity and noise equivalent power (NEP) obtained in this work were 1956.72 V/W and 2.3753 pW/Hz^1/2^, respectively, which shows better performance than previously reported graphene-based SSD full-wave bridge rectifier and makes it a good candidate as a full-wave bridge rectifier.

## Figures and Tables

**Table 2:** Parameter values for each structural parameter of channel capacitances, *C_SSD_* and trench capacitance, *C_trench_*.

Capacitance	Applied Equation No	Parameter Values
*C_SSD_*	4	*a* = 0.115 µm, *b* = 0.215 µm, *c* = 0.715 µm
	6	*s* = 2.0 µm, *d* = 0.10 µm
*C_trench_*	4	*s* = 1.63 µm, *d* = 0.10 µm
	5	*s* = 2.0 µm, *d* = 3.40 µm

## Data Availability

Not applicable.
